# Determination of the size parameters of α-synuclein amyloid precursor forms through DLS analysis

**DOI:** 10.1007/s00249-025-01737-z

**Published:** 2025-03-03

**Authors:** Marco A. Saraiva

**Affiliations:** 1 Centro de Química Estrutural, Institute of Molecular Sciences, Instituto Superior Técnico, University of Lisbon, Av. Rovisco Pais, Campus Alameda, 1049-001 Lisbon, Portugal; 2 Instituto de Tecnologia Química e Biológica António Xavier, Universidade Nova de Lisboa, Av. da República, 2780-157 Oeiras, Portugal

**Keywords:** α-Synuclein, Dynamic light scattering, Autocorrelation function, Amyloid precursor forms, Aggregate length, Width and height

## Abstract

Currently, there is an increased interest in identifying the characteristics of amyloid aggregates in the initial stages of amyloid formation. The aggregation mechanism of the α-synuclein (Syn) amyloid protein, which has been extensively studied, is still not fully understood. I show that with conventional dynamic light scattering (DLS) technique, the measurements of the dimensions of Syn amyloid precursor forms can be done early in the protein incubation. Additionally, the early aggregation of the Syn protein was initially studied by analyzing autocorrelation functions from fit distributions up to 10^4^ µs in the initial DLS measurements, specifically within the first 21 min. Investigation was conducted on the variation in the pH of the Syn solution throughout time. Based on DLS data, large Syn aggregated species formed from the monomer protein species. Afterward, I generated the autocorrelation functions based on the original DLS data, extending the fit distributions up to 10^5^ µs and noticed the existence of elongated Syn amyloid precursor forms in the protein solutions. Because the length of the elongated Syn amyloid precursor forms closely matches the wavelength of the incident light, the combination of translational diffusion *D*t and rotational diffusion *D*r in the decay rates enabled the measurement of their geometric dimensions through DLS. The improved precision of the fitted distributions I offered resulted in a new interpretation for the Syn protein aggregation in the initial stages. Overall, the methodology used in this study could be an effective strategy for examining how Syn amyloid precursor forms develop over time.

## Introduction

The process of disordered peptides and proteins spontaneously forming amyloid fibrils can be described as a type of nucleated growth polymerization pathway (Ferrone 1999; Wetzel 2006). In this process, the rate at which amyloid is formed is mainly influenced by the slow creation of nuclei in the beginning, which then grow quickly through monomer addition to the ends of the fibrils (Ferrone 1999; Wetzel 2006). In numerous amyloid systems, the process is made more complex by the initial development of different noncovalent oligomeric forms (Wetzel 2006; Harper et al. 1997), but basic polyglutamine peptides do not seem to create these structures in their natural state, enabling the study of both stages of amyloid growth to be relatively simple (Wetzel 2006). The aggregation nucleation thermodynamic model views the nucleus as the least stable identifiable species on the aggregation pathway, existing in a pre-equilibrium with ground-state monomers, contributing to both the equilibrium constant for nucleus formation and the rate constant for nucleus elongation in the nucleation rate (Ferrone 1999; Wetzel 2006). In this scenario, after being created, the nucleus can either return to its original state or go through a number of elongation stages that make the system more stable. As elongation can occur effectively from both natural nuclei and external seeds, studying seeded fibril growth allows for the isolated investigation of the elongation process. This is especially valuable for deciphering nucleated growth kinetics parameters and researching fibril formation reactions where the nucleation step is unclear or intricate, like in Aβ peptide-mediated Alzheimer's plaque fibril formation (Wetzel 2006). Regarding the protein being examined in the present study, the recent research suggests that the elongation process, when analyzed in the presence of fibrillar seeds, includes a secondary nucleation process (Xu et al. 2024). This protein is the α-synuclein (Syn) amyloid protein, linked to the development of Parkinson's disease. During the secondary nucleation, the kinetic models show that fibrils promote the creation of new fibrils (Xu et al. 2024). Additionally, analyzing fibril length suggests that secondary nucleation is the primary mechanism for fibril formation, rather than fibril fragmentation (Xu et al. 2024). In other words, the latest evidence suggests that Syn aggregates are created through secondary nucleation, rather than basic primary nucleation, and agitation boosts this process (Xu et al. 2024). Furthermore, by utilizing a combination of single molecule and bulk level methods, researchers discovered that secondary nucleation on existing fibril surfaces, rather than direct formation from monomers, is the primary source of oligomers (Xu et al. 2024). As a result, it appears that secondary nucleation is not just the primary origin of oligomers, but also the primary process behind Syn aggregate formation. The straightforward nucleation and elongation routes above referred alone may not fully explain the mechanisms of Syn aggregate formation, but there is evidence suggesting that Syn amyloid fibrils and their precursor forms may be involved in the overall aggregation pathway of Syn. Furthermore, it has been reported that the formation of protein condensates through liquid–liquid-phase separation (LLPS) can have an impact in the initial stages of Syn protein aggregates’ development (Dada et al. 2024; Piroska et al. 2023), namely the significance of inhibiting of the primary nucleation microscopic step within the condensates (Dada et al. 2024) and the early interplay between Syn amyloid fibrils and protein condensates formation (Piroska et al. 2023). In this regard, I opted to study the initial 21 min of Syn protein aggregation using dynamic light scattering (DLS). DLS is a powerful method that relies on the movement of particles caused by laser sources. DLS can quickly give details on the size of particles in solution and analysis of intensity-based autocorrelation functions can also offer insight into the Syn aggregation process. It is anticipated that the Syn protein solution will be initially populated by large aggregates (Saraiva 2020, 2021; Saraiva and Florêncio 2022), thereby highlighting the usefulness of DLS in studying amyloid systems. Additionally, it remains to be determined which of the previously mentioned mechanisms, i.e., nucleated growth polymerization pathway or the formation of protein condensates through LLPS, leads to the development of the aforementioned large Syn aggregates.

Moreover, DLS is commonly employed to assess the purity of Syn samples during protein purification and is often used to characterize Syn species isolated through chromatographic techniques. DLS necessitates protein samples with low polydispersity due to its high sensitivity. Therefore, DLS is not preferred for analyzing protein samples in the final stage of Syn protein aggregation, such as for identifying mature Syn amyloid fibril features. In our prior research, we found that early large Syn aggregates can be detected by DLS, but they represent less than 0.01% of all Syn species in solution (Saraiva 2020, 2021; Saraiva and Florêncio 2022). Therefore, there was a difference in concentration of four orders of magnitude between the early large Syn aggregates and the Syn monomers. Similarly, a starting Syn monomer concentration of 33.5 µM (0.5 mg/mL) is equivalent to approximately 10^−9^ to 10^−10^ M for early sizable Syn aggregates (a difference of four orders of magnitude) (Saraiva 2020, 2021; Saraiva and Florêncio 2022). Also, in the previous studies, we were unable to ascertain the type of early large Syn aggregates, because DLS software inaccurately identified the protein species in the initial stages of Syn protein incubation, such as in the initial 3 min (Saraiva 2020, 2021; Saraiva and Florêncio 2022). In this report, I first conducted static light scattering (SLS) experiments to determine which of the earlier referred mechanisms, namely the nucleated growth polymerization pathway or the formation of protein condensates through LLPS, leads to the development of early large Syn aggregates already detected by DLS in the previous studies (Saraiva 2020, 2021; Saraiva and Florêncio 2022). Second, I employed the DLS method to examine the development of large Syn protein aggregates in the initial 21 min of the protein incubation. During the DLS experiments, the initial measurements, i.e., particularly the initial 3 min, lacked a precise distribution fit, which was attributed to the DLS program, as stated. In this regard, I utilized autocorrelation functions from the DLS measurements obtained within the initial 21 min to predict the tendency for Syn aggregation under various protein solution conditions, specifically in the pH range from 7 to 2. Next, I created a mathematical interpretation utilizing the sum of the exponentials to simulate the autocorrelation functions acquired particularly from the initial DLS measurements, i.e., the initial 21 min with particular emphasis on the initial 3 min. The outcome was the identification of the size of the previously mentioned large Syn aggregates in the protein solutions, and it was also established that these are elongated Syn amyloid precursor forms present in the protein solutions. DLS was used to determine the geometric dimensions of the referred elongated Syn amyloid precursor forms by comparing their inferred length to the incident light wavelength, which is linked to both translational diffusion *D*t and rotational diffusion *D*r (Alexander-Katz 2013). To achieve this objective, analyzing the autocorrelation functions from the initial DLS measurements helped to better understand how the elongated Syn amyloid precursor forms evolve in terms of both length and width. Different scenarios of the protein solution were examined at this stage, focusing on how the solvent molecules around the amyloid fibril width impact translational diffusion perpendicular to the small elongated Syn amyloid precursor forms axis (height measurement).

## Methods

### α-Synuclein expression and purification

The pT7-7 plasmid containing the human Syn sequence (kindly provided by Professor Doctor T. Outeiro, IMM, University of Lisbon) was used to overexpress Syn in *Escherichia coli* BL21(DE3) bacteria. Syn was purified as previously described (Saraiva 2020, 2021; Saraiva and Florêncio 2022).

### Static light scattering (SLS)

Static light scattering was recorded using an SPEX Fluorolog 212I spectrofluorometer. Time scans of 1000 s were collected in the S/R mode and by setting both the excitation and emission monochromators at 275 nm. Quartz fused cuvettes with 5 mm path length were used.

### Dynamic light scattering (DLS)

Particle diffusion coefficients were measured by DLS using a Zetasizer Nano ZS device [He–Ne Red laser (633 nm), Malvern Instruments] as previously described (Saraiva 2020, 2021; Saraiva and Florêncio 2022). It should be noted that the particle diffusion coefficients were derived from analyzing the obtained intensity autocorrelation function. In addition, the Malvern general purpose algorithm was used, which indicates an α parameter or “regularizer” of 0.01. Accordingly, the indicated α parameter is best suited for the analysis of protein samples using Malvern instruments, as tested for lysozyme (0.3 mg/mL in PBS buffer at pH 6.8) and denatured hemoglobin at 44 °C (PBS buffer at pH 6.8). A Syn protein concentration of 33.5 μM (*A*_275nm_ = 0.2; 0.5 mg/mL) at a solution pH of 7 was optimized for DLS experiments to ensure that instrument count rates were in the range of 200–500 kcps (kilo counts per second) (Saraiva 2020, 2021; Saraiva and Florêncio 2022). All the materials used were carefully cleaned and manipulated to avoid dust particles, and the solutions of buffer and diluted HCl were previously filtered with 0.22 µm pore filters. The Syn stock solutions used in the DLS measurements were filtered by centrifugation with a 100 kDa MWCO (molecular weight cut-off) membrane centrifuge filter (Amicon, the membrane was made of regenerated cellulose) [centrifugation conditions: 5000 rpm (3214*g*, relative centrifugal force (RCF)] at 4 °C for 3 min (1 mL protein stock sample), fixed-angle rotor, Eppendorf Centrifuge 5810). A simple protocol based on filtration through a 100 kDa MWCO membrane has been reported to provide an efficient method for producing an aggregate-free Syn preparation and for rapid removal or isolation of Syn oligomers (> Syn dimers) (Kumar et al. 2020). For sample preparation, 1 mL of protein solution in 10 mM Tris–HCl at a concentration of 33.5 µM at the desired pH (prepared with high-purity Milli-Q water) was carefully transferred to a quartz cuvette (Hellma Analytics—QS 10.00 mm), and the experiments were performed at 20.0 °C. The pH of the protein solution was adjusted prior to measurement. The number of DLS runs was automatic. The duration of each run was approximately 8 s. In addition, the number of runs was similar for each DLS measurement. The protein samples were equilibrated at 20.0 °C for 120 s, and the data were recorded every 2 min. A total of ten DLS measurements were obtained, being equivalent to 21 min of incubation time for a given protein solution tested. Additionally, after centrifugation of Syn stock solutions for the removal of possible aggregates or impurities that developed during protein purification and after protein concentration determination, the initial 33.5 µM Syn solution was equilibrated for 120 s (2 min) at 20.0 °C in the DLS instrument, as mentioned, in addition to the 60 s required for the DLS measurement. The Malvern records page mentions the latter time in question with regards to "Intensity PSD". Thus, 3 min was not the selected time but rather the dead time for obtaining the initial DLS measurement.

## Results and discussion

**Fig. 1:**
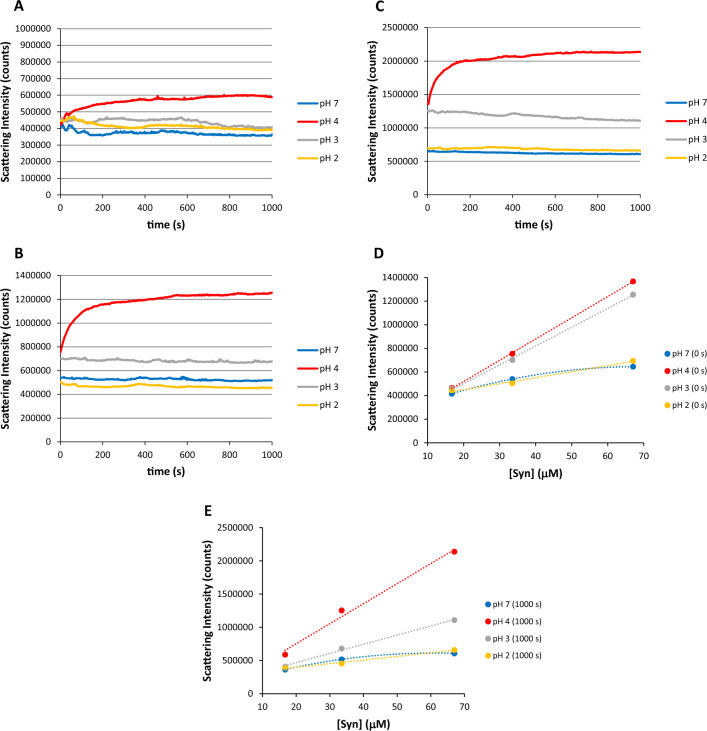
Static light scattering (SLS) data obtained for Syn solutions. **A** Rayleigh scattering intensity as a function of the time for a 16.7 µM Syn concentration (*A*_275nm_ = 0.1; *ε* = 5974 M^−1^ cm^−1^) at pH 7, 4, 3, and 2. **B** Rayleigh scattering intensity as a function of the time for a 33.5 µM Syn concentration (*A*_275nm_ = 0.2; *ε* = 5974 M^−1^ cm^−1^) at pH 7, 4, 3 and 2. **C** Rayleigh scattering intensity as a function of the time for a 67.0 µM Syn concentration (*A*_275nm_ = 0.4; *ε* = 5974 M^−1^ cm^−1^) at pH 7, 4, 3 and 2. **D** Recorded Rayleigh scattering intensity values at 0 s as a function of the Syn concentration at pH 7, 4, 3, and 2. **E** Recorded Rayleigh scattering intensity values at 1000 s as a function of the Syn concentration at pH 7, 4, 3, and 2. The time scans in **A**, **B**, and **C** were performed in triplicate and excitation and emission monochromators in the spectrofluorometer were both set at 275 nm. The recorded Rayleigh scattering intensity values are approximately tenfold greater than those previously reported (Saraiva and Florêncio 2022) due to the use of increased aperture of the machine slits. The Rayleigh scattering variations for the Syn protein concentrations of 16.7 and 33.5 µM at pH 7, 4, 3, and 2 are comparable to those reported (Saraiva and Florêncio 2022)

**Fig. 2:**
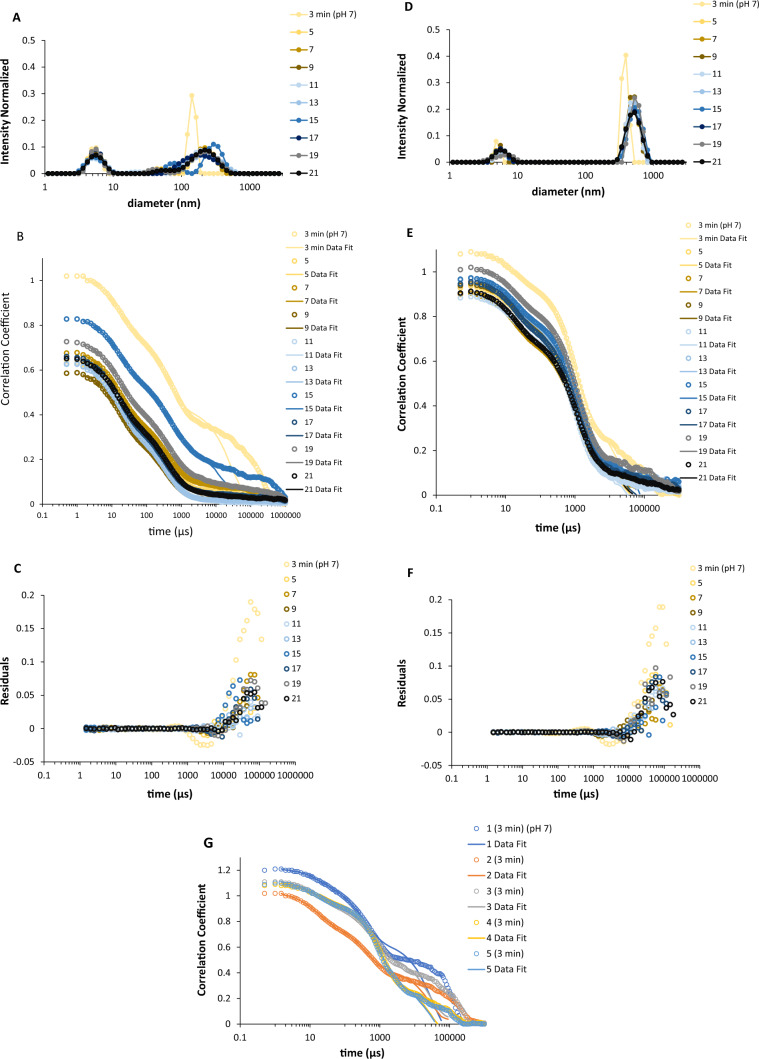
DLS analysis of Syn protein solutions at pH 7 over time. **A** Determined size distributions for ten DLS measurements (up to 21 min) for a 33.5 µM Syn protein concentration (*A*_275nm_ = 0.2; *ε* = 5974 M^‒1^ cm^‒1^; protein stock solution concentration of 102 µM) at 20.0 °C. **B** The obtained autocorrelation functions and corresponding fit distributions for **A**. **C** Residuals of **B**. **D** Determined size distributions for 10 DLS measurements (up to 21 min) for a 33.5 µM Syn protein concentration (*A*_275nm_ = 0.2; *ε* = 5974 M^‒1^ cm^‒1^; protein stock solution concentration of 125 µM) at 20.0 °C. **E** Obtained autocorrelation functions and corresponding fit distributions for **D**. **F** Residuals of **E**. **G** Obtained autocorrelation functions and corresponding fit distributions while varying the protein stock solution concentration: 83.7 µM (1), 102 µM (2), 116 µM (3), 125 µM (4), and 153 µM (5). In this figure, the duration of the DLS measurements is indicated

Syn protein (14.5 kDa) solutions were subjected to centrifugation filtration using a 100 kDa MWCO membrane centrifuge filter before SLS measurements (Fig. 1) were obtained. The small amounts of the already reported large Syn aggregates are caused by their early formation in Syn protein solutions after centrifugation, not by aggregates or impurities during protein purification. In the SLS experiments, I decided to investigate the Syn solutions at the selected pH values of 7, 4, 3, and 2 while varying the concentration of the protein. In the subsequent DLS experiments, additional pH values were studied within the range from 7 to 2. Also, examining the DLS data and observed that particularly the initial measurement, as previously mentioned, showed fewer wide peaks in the size distributions than did the following measurements (Fig. 2). Furthermore, the DLS analysis software (Malvern general purpose algorithm with an *α* parameter = 0.01 and quadratic weighting scheme) could not accurately fit the acquired autocorrelation functions for the initial DLS measurements. Therefore, it was initially examined these autocorrelation functions and assessed the possible aggregation tendency of the Syn solutions as the pH decreased from 7 to 2. Furthermore, a mathematical justification utilizing the cumulative sum of exponentials has been created to model the acquired autocorrelation patterns to ascertain the actual size of the transient spikes observed in the initial DLS measurement as well as in the following DLS measurements. By doing so, I can provide a clearer understanding of the DLS findings for the Syn protein.

### Static light scattering experiments to determine the mechanism underlying the formation of early large Syn protein aggregates in solution

Figure 1A–C shows the recorded Rayleigh scattering intensity (counts) as a function of the time for the protein concentrations of 16.7, 33.5, and 67.0 µM at the selected pH values of 7, 4, 3, and 2. It is clear from these figures that increased protein aggregation occurs at pH 4, as to be expected, whereas at pH 7, 3, and 2, minor aggregation occurs, i.e., considering the different protein concentrations investigated. At time 0 s and 1000 s, in particular, the recorded Rayleigh scattering intensity values retrieved from Fig. 1A–C at pH 7, 4, 3, and 2 as a function of the Syn protein concentration are depicted in Fig. 1D and E, respectively. From the latter figures, it is clear that the Rayleigh scattering intensity at both 0 s and 1000 s indicates linear trends at pH 4, 3, and 2 with the exception at pH 7 where there is a slight deviation from linearity. If protein condensates formation were to be exclusively formed at these different pH values examined, it would be expected significant deviations from linearity in Fig. 1D and E due to the protein saturation concentration *c*_sat_, that appears as a characteristic of the protein condensates formation (Alberti et al. 2019). Inspection of Fig. 1D and E reveals that no condensates formation arises at pH 4, 3, and 2, but one should not neglect possible condensates formation at pH 7. The minor deviation from linearity at pH 7 Syn solutions can be attributed to protein condensates formation, and, in fact, using similar solution conditions and by monitoring the ANS dye fluorescence intensity, we predicted such condensates formation at this pH value (Saraiva and Florêncio 2022). Overall, it can be inferred that mainly no Syn protein condensates formation occur at pH < 7 and, therefore, the nucleated growth polymerization pathway seems more likely to be the prevalent mechanism that better describes the Syn aggregation process in the protein solution conditions investigated in the present report.

### Analysis of the autocorrelation functions obtained by DLS when varying the concentration of the Syn protein solution

It is crucial to note that in Fig. 2, DLS measurements were carried out at a protein monomer concentration of 33.5 µM. Figure 2A shows the size distributions of 33.5 µM Syn protein (*A*_275nm_ = 0.2; *ε* = 5974 M^‒1^ cm^‒1^; protein stock solution concentration of 102 µM) at neutral pH and at 20.0 °C over time. This figure shows that the size distribution of the initial DLS measurement has narrower peaks than the peaks seen in the later size distributions: one peak represents the presumed Syn monomer species (average size: 5.34 nm, in diameter), and the other peak corresponds to an aggregated Syn species (average size: 145 nm, in diameter). Additionally, Fig. 2A shows wide peaks that represent the aggregated Syn species, indicating that a slow nucleation process is causing the aggregation of these protein species in the solution. Moreover, the wide peaks mentioned indicate the presence of diverse aggregated Syn species in the protein solution, meaning that protein species of very different sizes are present. Figure 2B displays the autocorrelation functions and fit distributions for the size distributions depicted in Fig. 2A. This already suggested that the fitted distributions from the DLS software were inaccurate and did not accurately represent the true particle-size distributions, particularly for the initial DLS measurement. Figure 2C displays the discrepancy in fit distributions for autocorrelation functions obtained by DLS from Fig. 2B. It is evident that the residuals are typically elevated, particularly for the extended decay periods of the initial autocorrelation functions obtained, leading to underestimation of larger protein particle sizes. Then, I conducted DLS measurements using an increased concentration of the protein solution (125 µM) (Fig. 2D). Figure 2D shows the size distributions obtained at a concentration of 33.5 µM for the Syn protein (*A*_275nm_ = 0.2; *ε* = 5974 M^‒1^ cm^‒1^; protein stock solution concentration of 125 µM) at neutral pH and 20.0 °C over time. Similar to Fig. 2A, Fig. 2D shows that the size distribution in the initial DLS measurement has also narrower peaks. One peak represents the potential Syn monomer species (average size: 5.00 nm, in diameter), and the other peak corresponds to the aggregated Syn species (average size: 386 nm, in diameter). The mentioned narrower peaks, which represent the aggregated Syn species, suggest that a rapid nucleation process is responsible for the aggregation of the Syn protein species in the solution. Thus, the narrower peaks noted in Fig. 2D suggest that there are fewer varied aggregated Syn species in the protein solution, which implies that there are less different sizes of protein species present, in comparison to what is shown in Fig. 2A. Similarly, to Fig. 2B, which reveals three exponentials in the decay times, Fig. 2E displays also three exponentials in the decay times. Figure 2E displays the autocorrelation functions and the fit distributions corresponding to the size distributions depicted in Fig. 2D. The autocorrelation functions and fitted distributions depicted in Fig. 2E exhibit similar accuracy in comparison to those in Fig. 2B for the initial DLS measurements. According to Fig. 2A, a more heterogeneous protein solution is expected, in contrast to the less heterogeneous protein solution observed in Fig. 2D. Furthermore, it was reported that the aggregation kinetics of Syn in the presence of 0.3 µM fibril seeds show that the protein aggregated faster at low concentrations than at higher protein concentrations (Meisl et al. 2022; Gaspar et al. 2017). In fact, it appears that Syn aggregation kinetics are independent of the protein monomer concentration at high concentrations (above approximately 30 µM) (Gaspar et al. 2017). It is important to note that these Syn experiments were performed in a mildly acidic pH (10 mM MES buffer at pH 5.5) and under quiescent conditions. Additionally, under such mildly acidic pH solution conditions, Syn protein aggregation is delayed at high concentrations due to a saturated secondary nucleation process (Gaspar et al. 2017). Because in Fig. 2A and D, protein stock solutions with concentrations of 102 and of 125 µM were used, which were subsequently diluted to the 33.5 µM for the DLS measurements, it appears that at the referred lower concentration (102 µM), Syn aggregates’ dissociation/disaggregation prevails over protein aggregation. This is reinforced in Fig. 2B, where the overall decrease in the amplitude of the autocorrelation functions suggests an increase in the protein aggregates dissociation/disaggregation (increased multiple scattering) due to the presence of a more heterogeneous protein sample. When using a more concentrated protein stock solution (125 µM), the amplitude of the autocorrelation functions remains relatively constant (due to reduced multiple scattering) (Fig. 2E), suggesting a less heterogeneous protein sample concomitant with the occurrence of minor aggregation in the protein solution, which prevails over the protein aggregates dissociation/disaggregation. Furthermore, when I contrasted the normalized intensity related to the Syn monomer species (Fig. 2A, D), the previous scenario is indeed supported. This means when using a less concentrated protein stock solution (102 µM), more protein monomer species are generated due to the protein aggregates dissociation/disaggregation, whereas when using a more concentrated protein stock solution (125 µM), fewer protein monomer species appear in solution due to the aforementioned minor protein aggregation occurring under such solution conditions. Hence, in this instance, the variation in the amplitude of the autocorrelation functions does predict the level of Syn protein aggregation in the system. Moreover, it has been reported that below the protein stock solution concentration of approximately 100 µM micellar-like aggregates are formed for the Syn solutions (Saraiva 2021). The fact that when using a protein stock solution concentration of 102 µM, it appears that the Syn aggregates dissociation/disaggregation prevails, which allows one to infer that the formation of the mentioned micellar-like aggregates for Syn can counteract with the event of the protein dissociation/disaggregation to favor this protein aggregation. In fact, when using a protein stock solution concentration of 125 µM, indeed, protein aggregation occurs. Another important aspect is that determined dissociation rate constants are low, in the order of 10^−4^ to 10^−5^ s^−1^ (Saraiva and Florêncio 2022). Therefore, when protein stock solutions are diluted to a protein concentration of 33.5 µM, there is no significant alteration in the amounts of protein aggregates present in the solutions for performing the DLS measurements. This means that the actual concentration of the protein stock solutions is the important factor for assessing the kinetics of the Syn aggregation process.

I also performed DLS measurements using several concentrations of the protein stock solution (Fig. 2G). In Fig. 2G, it is clear that lower protein stock solution concentrations contain more larger protein particles with longer decay times. Autocorrelation functions remained consistent for protein stock solution concentrations of at least 125 µM. Therefore, I decided to use protein stock solutions with concentrations of 125 µM or higher for the DLS measurements. The explanations are: (1) the stability of autocorrelation functions at pH 7 for better reproducibility, (2) lower heterogeneity of protein particles in the solution, and (3) enhanced fitting distributions for autocorrelation functions.

### DLS analysis by varying the pH of the Syn protein solutions

**Fig. 3:**
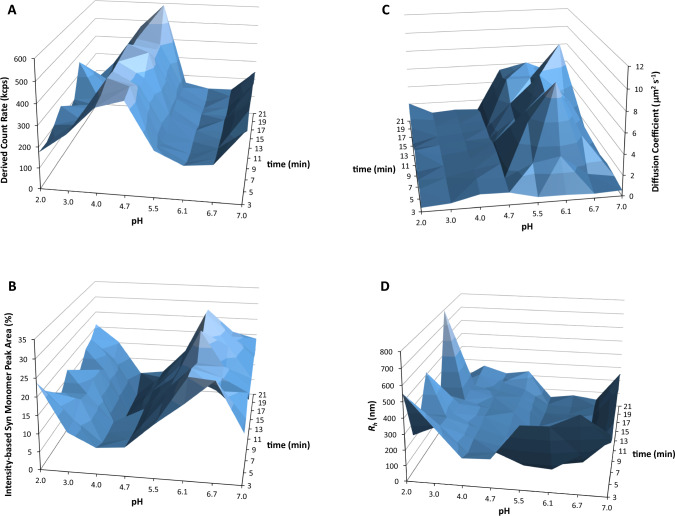
DLS analysis of Syn protein solutions with different pH levels ranging from pH 7 to pH 2 and varying incubation times. **A** Derived count rates (kcps) obtained as a function of protein solution pH in the pH range from 7 to 2 and for each pH value investigated ten DLS measurements (up to 21 min) were performed. **B** Intensity-based Syn monomer peak area (%) obtained as a function of protein solution pH in the pH range from 7 to 2 and for each pH value investigated ten DLS measurements (up to 21 min) were performed. **C** Diffusion coefficient obtained as a function of protein solution pH in the pH range from 7 to 2 and for each pH value investigated ten DLS measurements (up to 21 min) were performed. **D** Calculated *R*_h_ as a function of protein solution pH in the pH range from 7 to 2 and for each pH value investigated ten DLS measurements (up to 21 min) were performed. The displays were altered to enlarge their appearance

Figure 3A shows the derived count rates (kcps) obtained as a function of protein solution pH in the pH range from 7 to 2 and for each pH value investigated ten DLS measurements (up to 21 min) were performed. Protein stock solutions with concentrations ≥ 125 µM at a pH of 7 were utilized. It is evident from this illustration that the highest derived count values were associated with pH values of 4.7 and 4.0 (Fig. 3A). The pH values mentioned above also showed peak values in Rayleigh light scattering measurements conducted under similar protein solution conditions (Saraiva and Florêncio 2022). The highest expected aggregation of Syn will be at the protein's isoelectric point (p*I* = 4.7) rather than at pH 4.0, which implies that pH 5.5 and pH 4.0 are equally distant from the protein's isoelectric point, indicating that the protein aggregation at pH 4.0 does not stem from molecular effects at the isoelectric point (Fig. 3A). At a pH of 4.0, there may be partial protonation of aspartate and glutamate protein residues, potentially causing the Syn protein to undergo hydrophobic collapse to some degree. The hydrophobic collapse of the Syn protein involves the neutralized negatively charged C-terminal region associating with the hydrophobic NAC region of the amyloid protein. Nevertheless, at pH levels of 3.0 and 2.0, although the protein experiences complete hydrophobic collapse, the aggregation of Syn is not intensified as compared to pH 4.0 (Fig. 3A). It could be argued that raising the ionic strength and the consequently minimization of the electrostatic repulsions in the protein solution at pH levels of 3.0 and 2.0 may enhance the flexibility of the hydrophilic and exposed N-terminus (Saraiva 2020), consequently reducing Syn protein aggregation. Furthermore, it has been documented in literature that the aspartate and glutamate protein residues exhibit lower acidity in the C-terminus compared to the N-terminus of the Syn protein (Croke et al. 2011). As a result, pH 4.0 may trigger the beginning of protein hydrophobic collapse, promoting Syn protein aggregation. The reason for heightened Syn protein aggregation at pH 4.0 is due to the slight intermolecular effects happening at this pH, along with the anticipated partial hydrophobic collapse of the protein, which can promote Syn aggregation. In general, the predominant factor leading to the highest Syn aggregation at pH 4.7 and pH 4.0 seems to be primarily the intramolecular protein effects. Additionally, Fig. 3A shows that the aggregation of Syn protein is higher at pH 7.0 compared to the protein solution's pH levels up to 5.5, which are mildly acidic. Figure 3B shows the percentage of intensity-based monomer peak area obtained from DLS analysis. The variation is actually opposite to the derived count rates shown in Fig. 3A. This situation reveals that Syn protein monomer species primarily play a role in creating initial Syn aggregates. Moreover, I examined into the average diffusion coefficients obtained from the DLS analysis that includes less-accurate fit distributions of the autocorrelation functions acquired (Fig. 3C). The calculated average diffusion coefficients comprise the diffusion of both the initial Syn aggregates and the Syn monomer species in the protein solutions. Indeed, the data in Fig. 3C show that the diffusion coefficients are elevated in the slightly acidic range where Syn protein aggregation is minor (Fig. 3A), with values up to 10 × 10^−12^ m^2^ s^−1^, slightly less than the diffusion coefficients seen in small globular proteins, which are up to 10 × 10^−11^ m^2^ s^−1^ (Krouglova et al. 2004). This implies that the DLS average diffusion coefficients measured in the slightly acidic range up to pH 5.5 primarily reflect the diffusion of the Syn monomeric protein, which moves slower than compact proteins due to its disordered molecular structure in solution. In the region with a pH below 5.5, the diffusion coefficients measured reach up to 1 × 10^−12^ m^2^ s^−1^, which is ten times lower than those reported in the slightly acidic region up to pH 5.5. Increased protein aggregation is expected in the region with a pH below 5.5, as shown in Fig. 3A, which suggests that the lower diffusion coefficients mentioned above are related to the initial formation of Syn aggregated species in the protein solutions. The hydrodynamic radius (*R*_h_) was calculated using the measured diffusion coefficients and the Stokes–Einstein equation. In Fig. 3D, the variation in the calculated *R*_h_ is mainly opposite to the measured diffusion coefficients (Fig. 3C), as predicted by the Stokes–Einstein equation. The higher *R*_h_ measured at pH 2, as shown in Fig. 3D, may be attributed to the enhanced solubility of Syn aggregated species formed at this acidity level, potentially resulting from the increased flexibility of the unreacted N-terminus in the initial protein aggregates molecular structures (Saraiva 2020). The analysis of Fig. 3C and D was necessary, because the data involved accurately matched distributions up to 10^4^ µs of acquired autocorrelation functions, as shown in Fig. 2E. Then, I presented more precise fit distributions (by simulation) for autocorrelation functions obtained with decay times up to 10^5^ µs.

### Modeling the autocorrelation functions acquired through DLS

Before, I examined DLS data related to corresponding fit distributions up to 10^4^ µs of the autocorrelation functions obtained. Furthermore, the autocorrelation functions showed three intersecting curves, suggesting the existence of three exponential components. Then, I proceeded to the second approach in this study, which encompasses the development of a mathematical model to mimic the autocorrelation functions up to 10^5^ µs. This was done to improve the accuracy of fit distributions of the autocorrelation functions obtained and potentially identify the true size of the initial Syn aggregated molecular species in the protein solutions. DLS quantifies fluctuations in light intensity over time by measuring a second-order correlation function, *g*^(2)^ (*τ*). The intensity function is adjusted by a delay time (*τ*), and then, the autocorrelation function *g*(*τ*) is computed. The correlation function mentioned for a monodisperse sample can be expressed through the following equation:

1 $$g^{\left( 2 \right)} \left( \tau \right) = 1 + \beta \exp \left( { - 2\Gamma \tau } \right),$$

where *β* is the correlation function at zero delay, $\Gamma$ s the correlation function decay rate, and the baseline of the correlation function relaxes to a value of 1 at infinite delay. The correlation function decay rate, $\Gamma$, can be converted to the translational diffusion coefficient, *D*_t_, for the particle through the following relation:

2 $$D_{{\text{t}}} = \frac{\Gamma }{{q^{2} }}.$$

Thus, *q* is the magnitude of the scattering vector and is given by

3 $$\left| q \right| = \frac{{4\pi n_{0} }}{{\lambda_{0} }} \sin \left( {\frac{\theta }{2}} \right),$$

where *n*_0_ is the solvent index of refraction, *λ*_0_ is the vacuum wavelength of the incident light, and *θ* is the scattering angle. Additionally, the diffusion coefficient, *D*_t_, can be interpreted as the hydrodynamic radius, *R*_h_, of a diffusing sphere via the Stokes–Einstein equation

4 $$R_{{\text{h}}} = \frac{{k_{{\text{B}}} T}}{{6\pi \eta D_{{\text{t}}} }},$$

where *k*_B_ is the Boltzmann constant (1.381 × 10^‒23^ J K^‒1^), *T* is the temperature in Kelvin, and *η* is the absolute (or dynamic) viscosity of the solvent.

As mentioned above, the second-order correlation function *g*^(2)^(*τ*) can be related to the autocorrelation function *g*(*τ*) through the following expression:

5 $$g^{(2)} \left( \tau \right) - 1 = g\left( \tau \right) = \beta \exp \left( { - 2\Gamma \tau } \right).$$

The aforementioned explanation is applicable to a monodisperse sample examined using DLS. However, in the case of polydisperse samples such as the Syn solutions analyzed by DLS, the autocorrelation function is the result of multiple exponential decays within it. Hence, considering the presence of three exponentials in the autocorrelation functions in the current system, Eq. (6) can be adjusted as follows:

6 $$g \left( \tau \right) = \beta \exp \left( { - 2\Gamma_{1} \tau } \right) + \beta \exp \left( { - 2\Gamma_{2} \tau } \right) + \beta \exp \left( { - 2\Gamma_{3} \tau } \right).$$

In addition, due to the definition of *β,* I further normalized all correlation functions obtained to units; therefore, Eq. (6) is now given by

7 $$g\left( \tau \right) = \frac{{\left( {1 \times \exp \left( { - 2\Gamma_{1} \tau } \right) \times a_{1} + 1 \times \exp \left( { - 2\Gamma_{2} \tau } \right) \times a_{2} + 1 \times \exp \left( { - 2\Gamma_{3} \tau } \right) \times a_{3} } \right)}}{{a_{1} + a_{2} + a_{3} }}.$$

**Fig. 4:**
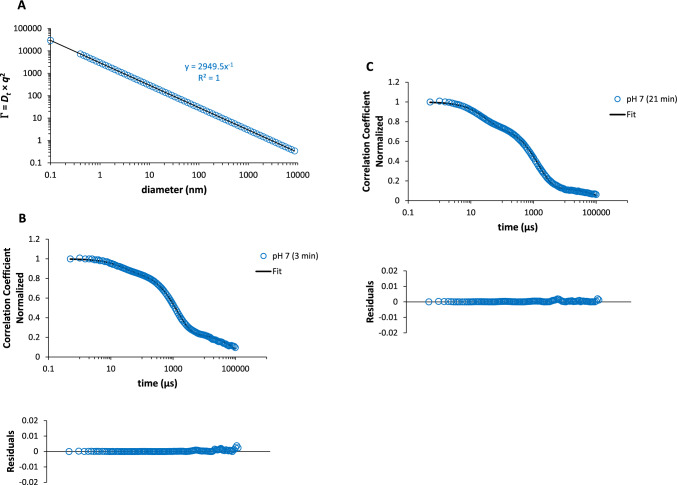
Simulation of the normalized autocorrelation functions for decay times up to 10^5^ µs. **A** Calculated decay rates, $\Gamma$, through the application of Eqs. (2), (3), and (4) as a function of the particle diameter (nm). Normalized autocorrelation functions for the Syn solution at pH 7 in the initial **B** 3 min and **C** 21 min and corresponding simulations (fits) due to the application of Eq. (7). The residuals of the referred simulations are indicated below

Therefore, Eq. (7) allows for the simulation of autocorrelation functions with decay times up to 10^5^ µs for the Syn solutions under DLS conditions, enabling the extraction of decay rates ($\Gamma_{1}$, $\Gamma_{2}$, and $\Gamma_{3}$) and exponential coefficients (*a*_1_, *a*_2_, and *a*_3_) for each autocorrelation function related to the Syn molecular species in the solution. It is crucial to note that the hydrodynamic diameter *d*_h_ (*d*_h_ = 2*R*_h_) of the Syn particles in solution in the simulated normalized autocorrelation functions was determined from the calculated decay rates, as mentioned above, and these decay rates were obtained through interpolation for single scatterers (Fig. 4A). As an example, in Fig. 4B and C, I show the normalized autocorrelation functions for the Syn solution at pH 7 for different incubation times, along with the simulations (fits) and corresponding residuals. The autocorrelation functions, normalized, represent the total of three exponentials, which utilized Eq. (7). As an illustration, Table 1 summarizes the results from using Eq. (7) on the normalized autocorrelation functions from the autocorrelation functions presented in Fig. 2E for the Syn solution at pH 7 over time (up to 21 min). The computed normalized autocorrelation functions for the Syn solutions accurately matched the autocorrelation functions of larger Syn particles acting as singular scatterers. Thus, the sizes of the Syn particles listed in Table 1 represent the aforementioned larger Syn particles in the solution at pH 7, as an example, and further investigation into their composition is warranted. The Chi-square (*χ*^2^) values of fit distributions shown in Table 1 are, in fact, low. When calculating the *p* values using the Chi-square scores, with degrees of freedom defined as (*N*_columns_ − 1) × (*N*_rows_ − 1) and a significance level of 0.05, the resulting *p* values are higher than the chosen level of significance (Chi-square test of independence). It is not the dependence of particles parameters with time that seems unfounded; the parameters determined by Eq. (7) are independent. The simulation provided using Eq. (7) is important, because these parameters are not related.

**Table 1:** Normalized exponential coefficients (*a*_1_, *a*_2_ and *a*_3_), decay rates ($\Gamma_{1}$, $\Gamma_{2}$, and $\Gamma_{3}$), and determined hydrodynamic diameters (*d*_h1_, *d*_h2_, and *d*_h3_) for the heavier Syn particles in solution determined by the application of Eq. (7) to the normalized autocorrelation functions for the autocorrelation functions depicted in Fig. 2E for the Syn solution at pH 7 over time (up to 21 min)

pH 7 (measurement in min)	*a*_1_	*a*_2_	*a*_3_	$\Gamma_{1}$	$\Gamma_{2}$	$\Gamma_{3}$	*d*_h1_ (nm)	*d*_h2_ (nm)	*d*_h3_ (nm)	*χ*^2^ (fit)
3	0.12	0.64	0.24	20,234	374.50	5.4695	0.15	7.9	539	0.036
5	0.19	0.68	0.13	16,836	364.92	3.9786	0.18	8.1	741	0.022
7	0.25	0.63	0.12	16,836	319.90	6.5163	0.18	9.2	453	0.087
9	0.18	0.67	0.15	16,836	379.17	5.2106	0.18	7.8	566	0.038
11	0.21	0.69	0.10	16,836	365.93	6.3844	0.18	8.1	462	0.041
13	0.20	0.66	0.14	16,836	353.96	3.8684	0.18	8.3	762	0.037
15	0.20	0.64	0.16	16,836	301.46	4.5310	0.18	9.8	651	0.056
17	0.22	0.64	0.14	16,836	353.96	3.4524	0.18	8.3	854	0.031
19	0.18	0.65	0.17	16,836	324.36	2.8880	0.18	9.1	1021	0.022
21	0.24	0.63	0.13	16,836	336.76	4.3796	0.18	8.8	673	0.036

The *χ*^2^ values are also indicated

The hydrodynamic diameters calculated for the heavier Syn particles in solution at pH 7 over time (Table 1), denoted as *d*_h1_, *d*_h2_, and *d*_h3_, suggested the movement of a lone scatterer under DLS conditions. According to the values of *d*_h3_ and *d*_h2_, this scatterer might be an elongated Syn species present in the solution. By this explanation, I suggest that the elongated Syn species referred to could be Syn amyloid precursor forms found in Syn solutions, which are likely created following the centrifugation process and utilized to eliminate large aggregated Syn species or impurities produced during the protein purification stages. In the literature, when *qL* is much greater than 1, as observed in diluted solutions of thin rods whose length is similar to the wavelength, *D*t = Γ/*q*^2^ → *D*_┴_ + (*L*^2^/12) × *D*r (Alexander-Katz 2013). This indicates the relationship between *D*_‖_, *D*_┴_, and *D*r and the length *L* and width *d* of the rod (Alexander-Katz 2013). This means that translational diffusion *D*t is linked to rotational diffusion *D*r. As a result, for thin rods with a length similar to the wavelength of the incident light, all diffusion coefficients and their geometrical dimensions can be determined by DLS (Alexander-Katz 2013). Indeed, in the simulation presented for autocorrelation functions from the initial DLS measurements, the elongated Syn amyloid precursor forms closely match the wavelength of incoming light (633 nm), as shown by measurements such as *d*_h3_ in Table 1. Furthermore, accurately determining the length and width (geometrical dimensions) of elongated Syn amyloid precursor forms, as in our simulation of autocorrelation functions from the initial DLS measurement, can only be achieved due to the coupling of translational diffusion *D*t with rotational diffusion *D*r in the decay rates, as mentioned. To validate the DLS analysis performed here, it is important to detach three aspects. First, when using Syn stock solutions with a concentration ≥ 125 µM, there is the occurrence of protein aggregation, although it is minor. Second, the highly concentrated Syn stock solutions used (protein concentration ≥ 125 µM), when diluted to a 33.5 µM protein concentration, does not significantly alter the amounts of protein aggregates present in the original protein stock solutions. This occurs, because the dissociation rate constants previously determined are low, on the order of 10^−4^ to 10^−5^ s^−1^, and these are not much affected by the pH of the diluted Syn solutions used in the DLS measurements (Saraiva and Florêncio 2022). Third, the highly concentrated Syn stock solutions used (protein concentration ≥ 125 µM) are likely to be homogeneous, encompassing the presence of protein aggregates of similar sizes, which is advantageous when employing the DLS technique (Fig. 2E)—DLS provides an average measurement over an ensemble of particles, which may not capture the detailed structural variations present within the sample.

**Fig. 5:**
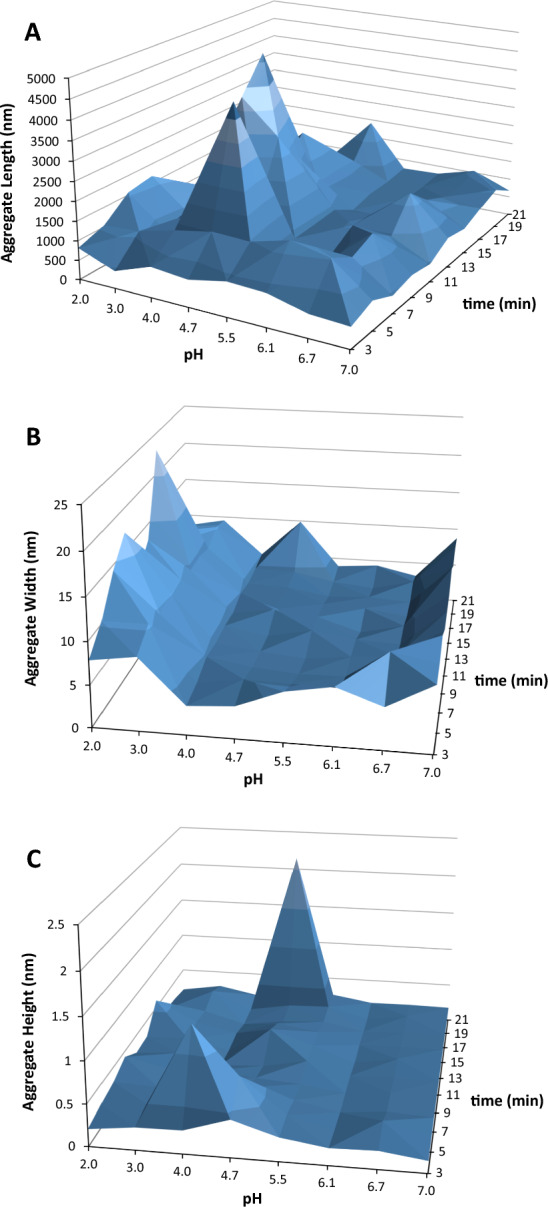
Aggregated Syn protein size parameters as a function of the protein solution pH from pH 7 to pH 2 over time (up to 21 min). **A** Syn aggregate length. **B** Syn aggregate width. **C** Syn aggregate height. The displays were altered to enlarge their appearance

Moreover, the outcomes presented in Table 1 are included in Fig. 5. In Fig. 5A, I show how the length of the elongated Syn precursor forms changes with the pH of the Syn protein solution, ranging from pH 7 to pH 2 over time (up to 21 min). It is evident from this figure that the length of the elongated Syn amyloid precursor forms (nm) slightly increases as the pH decreases from 7 to 5.5, then increases to pH 4.7 and 4.0, and eventually decreases again at pH 3 and 2. Syn protein aggregation was low at pH 6.7, pH 6.1, pH 5.5, pH 3, and pH 2 (Fig. 3A), and indeed, the lengths of the elongated Syn amyloid precursor forms (nm) decrease at these pH values in the protein solutions (Fig. 5A). Figure 5B displays the change in width (nm) of the Syn amyloid fibrils as a function of the pH of the Syn protein solution over time. This observed change in the width of the Syn amyloid precursor forms (nm) in Fig. 5B follows a pattern comparable to that of the change in the calculated *R*_h_ depicted in Fig. 3D. This means that the differences in the width of the Syn amyloid precursor forms (Fig. 5B) and the differences in the calculated *R*_h_ (Fig. 3D) actually show variations in how the decay time range used in the analysis of autocorrelation functions obtained by DLS is interpreted. The reported width of the Syn amyloid precursor forms (Fig. 5B) was determined with a tenfold longer decay time in the autocorrelation functions from DLS compared to the decay time used for calculating *R*_h_ (Fig. 3D). Moreover, in Fig. 5C, I show the previously discussed size component (*d*_h1_), possibly resulting from translational diffusion perpendicular to the Syn amyloid precursor forms small axis. The translational diffusion perpendicular to the axis of the Syn amyloid precursor forms helps to capture the movement of solvent molecules around the width of the fibrils. This movement could also retrieve the height of the Syn amyloid precursor forms (Fig. 5C). In addition, it is observed in Fig. 5B that the aggregation of Syn protein is more noticeable at pH 4.7 and 4.0 during various incubation durations. Also, in Fig. 5C, the height of Syn amyloid precursor forms changes depending on the incubation period of the protein solution, especially at pH levels of 4.7 and 4.0.

Currently, I can conclude that the intercept of the intensity autocorrelation functions for the first DLS measurement is mostly greater than 1 (Fig. 2B, E), in particular, which is a result of variations in the scattering intensity associated with two different movements of the elongated Syn amyloid precursor forms (rods) (Alexander-Katz 2013). The first is related to the translational motion of the rod, while the second dynamic mode is associated with the rotation of the rod around the center of mass of the rod (Alexander-Katz 2013). Moreover, it is anticipated that during the initial stages of aggregation, the newly formed Syn amyloid precursor forms do not form strong connections with Syn monomer species, resulting in increased translational and rotational movements. When the Syn monomer species interact more strongly with the Syn amyloid precursor forms, the translational and rotational movements of the Syn amyloid precursor forms (rods) likely decrease.

Importantly, due to the very low concentration of Syn amyloid precursor forms detected in this study (i.e., approximately 10^−9^–10^−10^ M), methods such as X-ray diffraction and solid-state NMR are not helpful for characterizing these species, as the signals observed are solely from the Syn monomer. This concentration is also much lower than the detection limits documented for atomic force microscopy (AFM) and transmission electron microscopy (TEM) (Ivanov et al. 2020; Rames et al. 2014). Reported protein concentrations in EM grids and AFM typically range from approximately 10^−7^ to 10^−8^ M (Ivanov et al. 2020; Rames et al. 2014). I believe that AFM and TEM cannot reveal the presence of the aforementioned Syn aggregated species. Moreover, at the estimated very low concentration of the Syn amyloid precursor forms (approximately 10^−9^–10^−10^ M), the thioflavin T dye does not detect these aggregated species in solution. The initial Syn protein monomers at a concentration of 0.5 mg/mL (33.5 µM) have a minimum lag phase time when the pH changes from 8.9 to 0.9, which is approximately 2 h (thioflavin T binding) (Uversky et al. 2001).

## Conclusions

In this study, I utilized a conventional DLS method to analyze the presence of Syn aggregated species during the initial stage of protein solution incubation up to 21 min. I focused primarily on analyzing the initial DLS measurements and found that the fit distributions from the DLS software did not accurately match the intensity autocorrelation functions I obtained. By analyzing the intensity autocorrelation functions and their corresponding fit distributions up to 10^4^ µs from the initial DLS measurements, I was able to determine the tendency of the Syn solutions to aggregate as the pH of the solution changed from 7 to 2 over time. Next, I modeled the autocorrelation functions providing to them fit distributions up to 10^5^ µs for the initial DLS measurements and specifically examined the properties of the large Syn aggregated species. In this simulation, large Syn species formed by aggregation were actually elongated Syn amyloid precursor forms present in the protein solutions. Because the elongated Syn amyloid precursor forms are similar in size to the wavelength of the incident light, DLS was able to calculate the geometric dimensions of the referred aggregated protein species by measuring the decay rates of translational diffusion *D*t and rotational diffusion *D*r combined. Moreover, the dimensions and the solvent volume surrounding the width of the elongated Syn amyloid precursor forms were measured under identical protein solution conditions to those previously mentioned. Modifying the pH of the protein solution over time caused the length and width of the Syn amyloid precursor forms to change in a different manner. With respect to determining the size of the solvent (scattering) surrounding the width of the elongated Syn amyloid precursor forms, both the former and length displayed comparable changes, serving as potential indicators of Syn protein aggregation in solution. Overall, the methodology implemented can be a useful approach for studying the development of Syn aggregated species in their early stages and determining their size parameters, such as length, width, and height.

## Data Availability

Data are available from the author upon request.
